# Near field effect on elasticity measurement for cartilage-bone structure using Lamb wave method

**DOI:** 10.1186/s12938-017-0417-9

**Published:** 2017-10-30

**Authors:** Hao Xu, Shigao Chen, Kai-Nan An, Zong-Ping Luo

**Affiliations:** 1 Orthopaedic Institute, Department of Orthopaedics, the First Affiliated Hospital, Soochow University, Suzhou, 215006 Jiangsu People’s Republic of China; 2 Department of Radiology, Mayo Clinic, Rochester, MN United States of America; 3 Biomechanics Laboratory, Division of Orthopedic Research, Mayo Clinic, Rochester, MN United States of America

**Keywords:** Elasticity assessment, Cartilage-bone structure, Lamb wave method, Near field effect

## Abstract

**Background:**

Cartilage elasticity changes with cartilage degeneration. Hence, cartilage elasticity detection might be an alternative to traditional imaging methods for the early diagnosis of osteoarthritis. Based on the wave propagation measurement, Shear wave elastography (SWE) become an emerging non-invasive elasticity detection method. The wave propagation model, which is affected by tissue shapes, is crucial for elasticity estimating in SWE. However, wave propagation model for cartilage was unclear.

**Methods:**

This study aimed to establish a wave propagation model for the cartilage-bone structure. We fabricated a cartilage-bone structure, and studied the elasticity measurement and wave propagation by experimental and numerical Lamb wave method (LWM).

**Results:**

Results indicated the wave propagation model satisfied the lamb wave theory for two-layered structure. Moreover, a near field region, which affects wave speed measurements and whose occurrence can be prevented if the wave frequency is larger than one critical frequency, was observed.

**Conclusion:**

Our findings would provide a theoretical foundation for further application of LWM in elasticity measurement of cartilage in vivo. It can help the application of LWM to the diagnosis of osteoarthritis.

## Background

Osteoarthritis is the most prevalent joint disease in senior people worldwide [1]. For end stage osteoarthritis, joint replacement surgery and pain management are the main clinical treatment options available due to the poor cartilage regeneration potential [2]. Therefore, early treatment, including preventing and even reversing the pathological changes of osteoarthritis, is desired. Fortunately, laboratory tests have partly verified the feasibility of early treatment [3]. Early treatment requires the early detection of osteoarthritis. However, early diagnosis of osteoarthritis cannot be achieved with traditional imaging diagnostic tools (i.e. X-ray and MRI) [4]. It is well established that the initial pathologic change observed in cartilage degeneration is focal glycosaminoglycan depletion. It changes the elastic properties of cartilage whereas the morphological appearance remains intact [5]. Previous studies have reported that a significant difference was observed in the surface elastic modulus of osteoarthritis (OA) grades 1 and 2 [6]. Therefore, compared to traditional imaging detection methods, elasticity detection may have the potential to early diagnose of osteoarthritis.

Shear wave elastography (SWE) is a newly developed non-invasive detection method based on elasticity detection [7, 8]. It has been widely used in the elasticity detection of various tissue lesions, such as liver fibrosis [7], breast lesions [7], and thyroid nodules [8]. However, commercial SWE is not acceptable for the elasticity diagnosis of cartilage owing to two major challenges. The first challenge is the difficulty of generation and measurement of mechanical waves in cartilage tissues. Commercial SWE machines can only detect tissues with Young’s modulus values less than 0.3 MPa [9, 10]. However, the Young’s modulus of cartilage exceeds the upper limit of commercial SWE machines [6]. In high stiffness tissues, mechanical wave motions generated by the ultrasound radiation force are too weak to be detected by the ultrasound probe. Therefore, a higher driving force and/or more sensitive measurement technology is required for cartilage elasticity measurement [7]. The second challenge is the appropriate wave propagation model in cartilage-bone structure. For tissue elasticity measurement with thin thicknesses (e.g. the myocardium [11] and arterial wall [12, 13]), the Lamb wave theory in single-layered structures is acceptable. Although the cartilage thickness is also thin, the Lamb wave propagation in a cartilage-bone structure is more complicated than that of myocardium because of the existence of the bone. Therefore, the Lamb wave propagation model in a cartilage-bone structure should be investigated.

The objective of this study is to develop the Lamb wave method (LWM) for elasticity measurement in cartilage-bone structure. Current study would provide a theoretical foundation for further application of LWM to cartilage in vivo. We fabricated a silicone-polypropylene plate to simulate the cartilage-bone structure. The lamb waves were generated by a mechanical shaker under different loading frequencies, and detected by a laser Doppler detector. The measured wave speed in the cartilage-bone structure was used to estimate silicone elasticity (simulated cartilage) using the Lamb wave propagation model. Numerical simulations were also carried out to deeply understand wave propagation in a cartilage-bone structure.

## Methods

### Simulated cartilage-bone structure preparation

To create an ideal model for the LWM test, a silicone-polypropylene plate was fabricated to simulate the actual cartilage-bone structure. The silicone plate was used to simulate the cartilage structure. A SYLGARD 184 silicone elastomer and a curing agent (Dow Corning, Midland, Michigan, USA) were mixed together in a mass ratio of 10:1 at room temperature. The mixture was poured into molds, degassed in a vacuum chamber for 20 min, and then maintained at 60 °C for 2 h to accelerate the curing process. The mold was removed when the silicone mixture was fully cured. From the mold, we obtained a silicone plate (0.8 cm × 20 cm × 20 cm). Then, the silicone plate was stuck to the polypropylene plate (3 cm × 20 cm × 20 cm) simulating the bone. Further, a cylinder silicone sample (diameter 19 mm and height 28 mm) and polypropylene sample (diameter 1.4 cm and height 3 cm) were fabricated for the uniaxial compression test. The measured density of the silicone and polypropylene samples were 1239 and 838 kg/m^3^, respectively.

### Uniaxial compression

A uniaxial compression test is the most commonly used material testing method. Hence, uniaxial compressions were performed to obtain the Young’s modulus of the silicone (simulated cartilage) and polypropylene (simulated bone) plate. The uniaxial compression test was performed three times to estimate the average Young’s modulus. The indenter moved at a rate of 0.1 mm/s to ensure quasi static compression. The force–deformation curves of the silicone and polypropylene samples were obtained by the uniaxial compression test. The relationship between the axial force F and the deformation δ is as follows:

1 $${\text{F}} = {\text{ES}}\frac{\delta }{\text{H}} ,$$

where *E* = 2(1 + *ν*)*G* is the Young’s modulus, *ν* is Poisson ratio and *G* is shear modulus. H is the sample thickness, and S is the contact area. The Poisson ratio of SYLGARD 184 ranges from 0.45 to 0.5 [14].

### Lamb wave theory in single and two-layered structure

It is well known that the wave propagation model is associated with the material properties and structure shape. For a single-layered isotropic structure embedded in air, wave propagation obeys the antisymmetric Lamb wave model [15], and is expressed as follows:

2 $$\frac{{{ \tan }\left( {\text{qh}} \right)}}{{{ \tan }\left( {\text{ph}} \right)}} = - \frac{{\left( {{\text{q}}^{2} - k^{2} } \right)^{2} }}{{4{\text{k}}^{2} {\text{pq}}}} ,$$

where $p^{2} = \left( {\omega /{\text{c}}_{L} } \right)^{2} - k^{2} ,\;\;q^{2} = \left( {\omega /{\text{c}}_{T} } \right)^{2} - k^{2} ,\; \, k = \omega /{\text{c}}$ is the wavenumber, ω is the angular frequency, c is phase velocity, c_L_ and ${\text{c}}_{\text{T}} = \sqrt {\mu /\rho }$ are longitudinal and transverse wave speeds, respectively, and 2 h is the thickness of the single layer structure. The frequency response of the bio-tissues could be modeled by Voigt model, so the shear modulus $\mu = \mu_{ 1} + i\omega \mu_{ 2} ,$ where *μ*_1_ and *μ*_2_ are the shear elastic and viscous modulus. In current study, the viscosity coefficient *μ*_2_ was ignored for the silicone and polypropylene samples. To obtain cartilage elasticity coefficient in pure cartilage structure, Eq. (2) is fitted to the Lamb wave dispersion curves (wave speed versus frequency).

However, wave propagation in a cartilage-bone structure is strongly influenced by the bone. It obeys the lamb wave theory for a two-layered structure. From the continuity of stresses and displacements across the interface between two adjacent elastic layers, the displacement–stress vectors of any two layers can be related as follows [16]:

3 $$\begin{gathered} \left\{ {\begin{array}{*{20}c} {u_{1} } \\ {u_{2} } \\ {\sigma _{{22}} } \\ {\sigma _{{12}} } \\ \end{array} } \right\}^{m} = \left[ {\begin{array}{*{20}c} {k_{{11}} } & {k_{{12}} } & {k_{{13}} } & {k_{{14}} } \\ {k_{{21}} } & {k_{{22}} } & {k_{{23}} } & {k_{{24}} } \\ {k_{{31}} } & {k_{{32}} } & {k_{{33}} } & {k_{{34}} } \\ {k_{{41}} } & {k_{{42}} } & {k_{{43}} } & {k_{{44}} } \\ \end{array} } \right]\left\{ {\begin{array}{*{20}c} {u_{1} } \\ {u_{2} } \\ {\sigma _{{22}} } \\ {\sigma _{{12}} } \\ \end{array} } \right\}^{n} = \hfill \\ ~~\left[ {\begin{array}{*{20}c} {g_{{11}}^{1} } & {g_{{12}}^{1} } & {g_{{13}}^{1} } & {g_{{14}}^{1} } \\ {g_{{21}}^{1} } & {g_{{22}}^{1} } & {g_{{23}}^{1} } & {g_{{24}}^{1} } \\ {g_{{31}}^{1} } & {g_{{32}}^{1} } & {g_{{33}}^{1} } & {g_{{34}}^{1} } \\ {g_{{41}}^{1} } & {g_{{42}}^{1} } & {g_{{43}}^{1} } & {g_{{44}}^{1} } \\ \end{array} } \right]\left[ {\begin{array}{*{20}c} {g_{{11}}^{2} } & {g_{{12}}^{2} } & {g_{{13}}^{2} } & {g_{{14}}^{2} } \\ {g_{{21}}^{2} } & {g_{{22}}^{2} } & {g_{{23}}^{2} } & {g_{{24}}^{2} } \\ {g_{{31}}^{2} } & {g_{{32}}^{2} } & {g_{{33}}^{2} } & {g_{{34}}^{2} } \\ {g_{{41}}^{2} } & {g_{{42}}^{2} } & {g_{{43}}^{2} } & {g_{{44}}^{2} } \\ \end{array} } \right]\left\{ {\begin{array}{*{20}c} {u_{1} } \\ {u_{2} } \\ {\sigma _{{22}} } \\ {\sigma _{{12}} } \\ \end{array} } \right\}^{n} , \hfill \\ \end{gathered}$$

where *u*_1_ and *u*_2_ are displacement, *σ*_12_ and *σ*_22_ are stress, and the superscript m and n are the layer number. The field matrix g can be found in the literature [16]. The coefficients of matrix g are:

4 $$\begin{gathered} g_{{11}} = \frac{{c_{T}^{2} k^{2} }}{{\omega ^{2} }}\left( {g_{L} + \frac{1}{{g_{L} }}} \right) + \frac{B}{{2\omega ^{2} }}\left( {g_{T} + \frac{1}{{g_{T} }}} \right)~~ \hfill \\ g_{{12}} = \frac{{kB}}{{2\omega ^{2} p}}\left( {g_{L} - \frac{1}{{g_{L} }}} \right) + \frac{{kc_{T}^{2} q}}{{\omega ^{2} }}\left( { - g_{T} + \frac{1}{{g_{T} }}} \right)~~ \hfill \\ g_{{13}} = \frac{k}{{2i\omega ^{2} \rho }}\left( {g_{L} + \frac{1}{{g_{L} }} -\, g_{T} - \frac{1}{{g_{T} }}} \right)~~ \hfill \\ g_{{14}} = \frac{{k^{2} }}{{2i\omega ^{2} \rho p^{2} }}\left( {g_{L} - \frac{1}{{g_{L} }}} \right) + \frac{q}{{2i\omega ^{2} \rho }}\left( {g_{T} - \frac{1}{{g_{T} }}} \right)~~ \hfill \\ g_{{21}} = \frac{{pc_{T}^{2} k}}{{\omega ^{2} }}\left( {g_{L} - \frac{1}{{g_{L} }}} \right) + \frac{{Bk}}{{2\omega ^{2} q}}\left( { -\, g_{T} + \frac{1}{{g_{T} }}} \right)~~ \hfill \\ g_{{22}} = \frac{B}{{2\omega ^{2} }}\left( {g_{L} + \frac{1}{{g_{L} }}} \right) + \frac{{c_{T}^{2} k^{2} }}{{\omega ^{2} }}\left( {g_{T} + \frac{1}{{g_{T} }}} \right)~~ \hfill \\ g_{{23}} = \frac{p}{{2i\omega ^{2} \rho }}\left( {g_{L} - \frac{1}{{g_{L} }}} \right) + \frac{{k^{2} }}{{2i\omega ^{2} \rho q}}\left( {g_{T} - \frac{1}{{g_{T} }}} \right)~~ \hfill \\ g_{{24}} = g_{{13}} ~~ \hfill \\ g_{{31}} = \frac{{i\rho Bc_{T}^{2} k}}{{\omega ^{2} }}\left( {g_{L} + \frac{1}{{g_{L} }} - g_{T} - \frac{1}{{g_{T} }}} \right) \hfill \\ g_{{32}} = \frac{{i\rho B^{2} }}{{2\omega ^{2} p}}\left( {g_{L} - \frac{1}{{g_{L} }}} \right) + \frac{{2i\rho c_{T}^{4} k^{2} q}}{{\omega ^{2} }}\left( {g_{T} - \frac{1}{{g_{T} }}} \right)~~ \hfill \\ g_{{33}} = g_{{22}} ~~ \hfill \\ g_{{34}} = g_{{12}} ~~ \hfill \\ g_{{41}} = \frac{{2i\rho c_{T}^{4} k^{2} p}}{{\omega ^{2} }}\left( {g_{L} - \frac{1}{{g_{L} }}} \right) + \frac{{i\rho B^{2} }}{{2\omega ^{2} q}}\left( {g_{T} - \frac{1}{{g_{T} }}} \right)~~ \hfill \\ g_{{42}} = g_{{31}} ~~ \hfill \\ g_{{43}} = g_{{21}} ~~ \hfill \\ g_{{44}} = g_{{11}} . \hfill \\ \end{gathered}$$

where $g_{L} = e^{iph} ,\;g_{T} = e^{iqh} \;{\text{and}}\;B = \omega^{ 2} - 2c_{T}^{ 2} k^{ 2} ,\;k = \omega /{\text{c}}$ is the wavenumber, ω is the angular frequency, c is phase velocity, c_L_ and ${\text{c}}_{\text{T}} = \sqrt {\mu /\rho }$ are longitudinal and transverse wave speeds, respectively. The frequency response of the bio-tissues could be modeled by Voigt model, so the shear modulus $\mu = \mu_{ 1} + i\omega \mu_{ 2}$. In current study, the viscosity coefficient *μ*_2_ was ignored for the silicone and polypropylene samples.

As both the half spaces were embedded in a vacuum in this test, the modal solution requires that the stresses be zero at the extreme interfaces; thus, we have

5 $$\left\{ {\begin{array}{*{20}c} {u_{1} } \\ {u_{2} } \\ 0 \\ 0 \\ \end{array} } \right\}^{m} = \left[ {\begin{array}{*{20}c} {k_{11} } & {k_{12} } & {k_{13} } & {k_{14} } \\ {k_{21} } & {k_{22} } & {k_{23} } & {k_{24} } \\ {k_{31} } & {k_{32} } & {k_{33} } & {k_{34} } \\ {k_{41} } & {k_{42} } & {k_{43} } & {k_{44} } \\ \end{array} } \right]\left\{ {\begin{array}{*{20}c} {u_{1} } \\ {u_{2} } \\ 0 \\ 0 \\ \end{array} } \right\}^{n} .$$

Expanding this equation for the two (zero) stress terms on the left hand side gives the following:

6 $$\left\{ {\begin{array}{*{20}c} 0 \\ 0 \\ \end{array} } \right\}^{m} = \left[ {\begin{array}{*{20}c} {k_{31} } & {k_{32} } \\ {k_{41} } & {k_{42} } \\ \end{array} } \right]\left\{ {\begin{array}{*{20}c} {u_{1} } \\ {u_{2} } \\ \end{array} } \right\}^{n} .$$

To satisfy this equation, the submatrix must be singular. Thus, the governing equation is

7 $$\left| {\begin{array}{*{20}c} {k_{31} } & {k_{32} } \\ {k_{41} } & {k_{42} } \\ \end{array} } \right| = 0.$$

When the determinant (Eq. 7) was expanded, we obtained

8 $$\left( {g_{31}^{1} g_{11}^{2} + g_{32}^{1} g_{21}^{2} + g_{33}^{1} g_{31}^{2} + g_{34}^{1} g_{41}^{2} } \right) \left( {g_{41}^{1} g_{12}^{2} + g_{42}^{1} g_{22}^{2} + g_{43}^{1} g_{32}^{2} + g_{44}^{1} g_{42}^{2} } \right) - \left( {g_{31}^{1} g_{12}^{2} + g_{32}^{1} g_{22}^{2} + g_{33}^{1} g_{31}^{2} + g_{34}^{1} g_{42}^{2} } \right) \left( {g_{41}^{1} g_{11}^{2} + g_{42}^{1} g_{21}^{2} + g_{43}^{1} g_{31}^{2} + g_{44}^{1} g_{41}^{2} } \right) = 0$$

To obtain cartilage elasticity coefficient in cartilage-bone structure, Eq. (8) is fitted to the Lamb wave dispersion curves (wave speed versus frequency).

### Experimental LWM tests

**Fig. 1:**
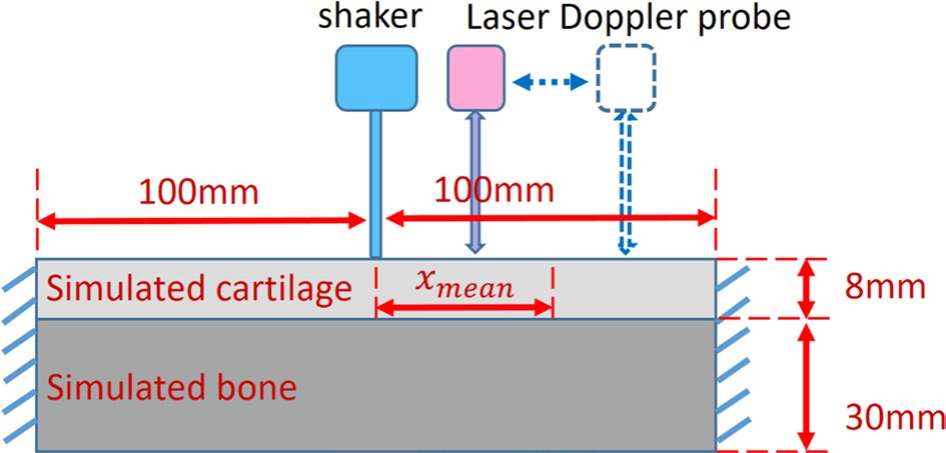
Setup for experimental LWM tests. The inputted sinusoidal voltage drove the mechanical shaker, in which a cylindrical bar was in contact with the sample surface. The particle velocities were detected by a Laser Doppler detector

Experimental LWM tests were carried out to study the elasticity measurement in the simulated cartilage-bone structure. The setup of the LWM experiment included the generating and measuring of Lamb waves (Fig. 1). A continuous sinusoidal input voltage was generated by the function generator (ATTEN ATF20B DDS) to drive the mechanical shaker (V201, BRUEL & KJAER, Denmark) at different frequencies ranging from 200 to 3000 Hz. The cylindrical bar (diameter 5 mm) mounted on the tip of the shaker was in contact with the sample surface. The particle velocities in the sample were detected with a laser Doppler detector system (Polytec PDV 100) and gathered by a digital oscilloscope (Rigol DS1102CA). The sinusoidal voltage wave from the function waveform generator was also gathered by the oscilloscope as the reference signal. Particle velocities were recorded at 11 points (0.5 cm apart) by shifting the laser detector along a line away from the cylindrical bar of the shaker. We defined the receiver distance *x*_*i*_ as the distance between the shaking point and the Doppler detector point, where the subscript i means the ith measurement. In the present tests, the receiver distances varied from 2.5 to 7.5 cm (0.5 cm increments). The mean receiver distance could be defined as

9 $$x_{mean} = mean(x_{i} )$$

Hence, the mean receiver distance in the experimental LWM tests was about 5 cm. Based on the particle velocity measured from different points, the wave speed could be estimated by the 2D fast Fourier transform (FFT) algorithm [11].

### Numerical LWM (finite element method)

A 2D axisymmetric finite element method (FEM) was used to investigate lamb wave propagation in the cartilage-bone plate. In the present study, the FEM package was the ABAQUS/CAE (SIMULIA, Providence, RI). The plate was represented with an axisymmetric element (CAX4R). The structure shape, material parameters, and the wave speed measurement method were established according to the experimental LWM tests described above. A fixed boundary condition was prescribed to the lateral surface of the cartilage-bone structure. A cyclic motion with a maximum displacement of 10 μm was applied to the middle of the cartilage surface as a point source. The cyclic motion had a loading frequency ranging from 200 to 3000 Hz.

## Results

**Fig. 2:**
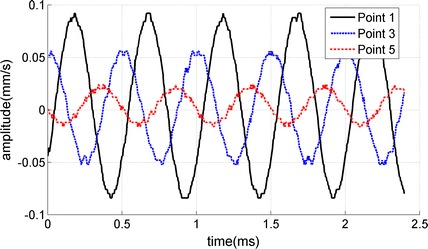
Relationship between the amplitude and time at point 1, 3 and 5 in experimental LWM tests (two layered structure, loading frequency is 2000 Hz, and the receiver distances x_i_ of point 1, 3 and 5 were 2.5, 3.5 and 4.5 cm respectively)

**Fig. 3:**
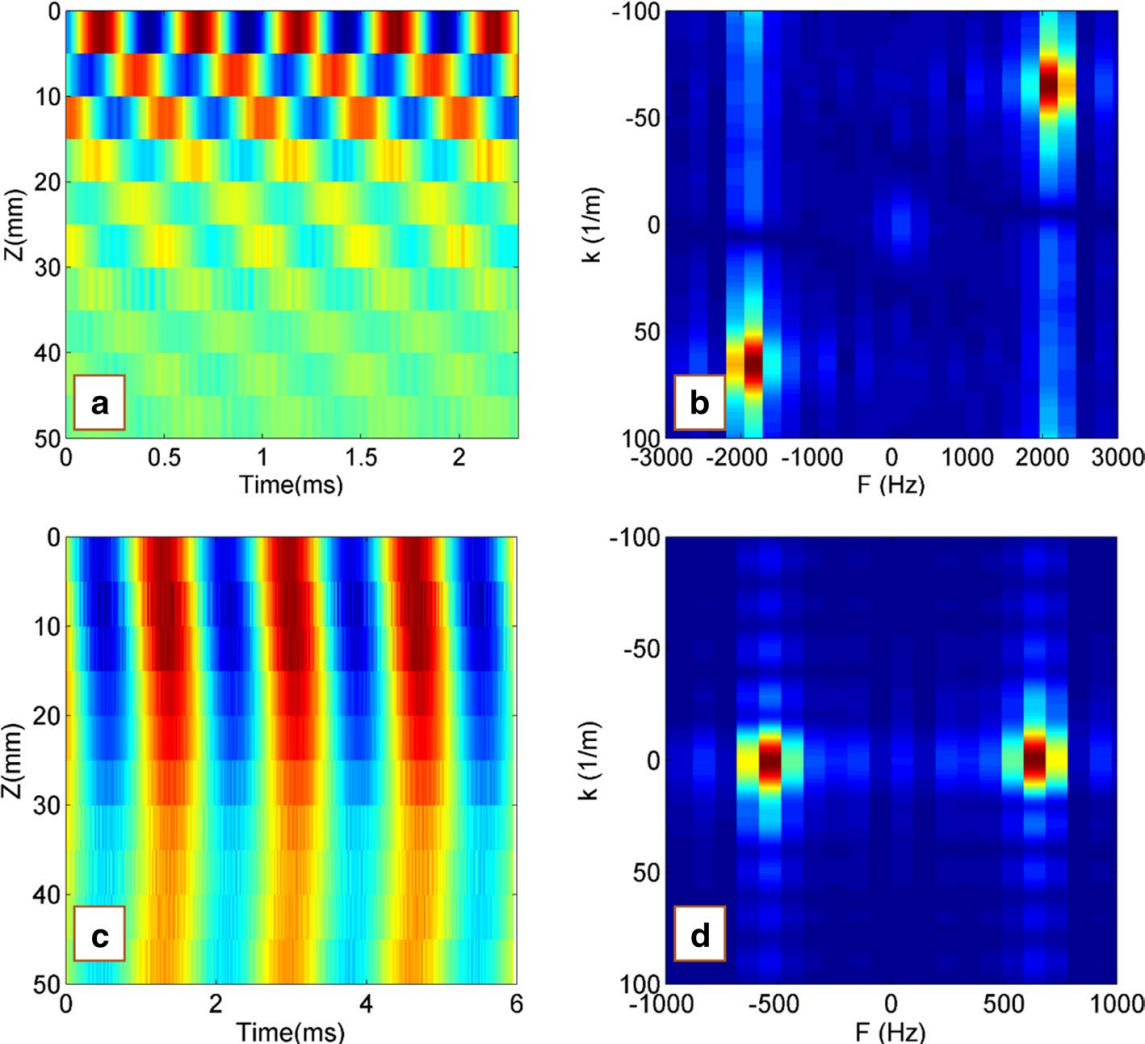
Experimental LWM results for the cartilage-bone structure: **a**, **c** velocity field of wave propagating and **b**, **d** the 2D FFT results. The wave speeds were estimated by dividing the frequency by the wave number at the peak energy (c = λf = f/k). **a**, **b** Loading frequency 2000 Hz, estimated wave speed 27 m/s; **c**, **d** loading frequency 600 Hz, estimated wave speed unknown

In the experimental LWM tests, under the driving force on the simulated cartilage-bone structure, the Lamb wave propagated from the driving point to the far field. As the receiver distance increased, amplitude and phase delay of the wave propagation were observed (Fig. 2). Using the measured particle velocity–time curves at different measuring points, we obtained the velocity field (typical motion map as a function of time and location) and the 2D FFT results (Fig. 3). The wave speeds were estimated by dividing the frequency by the wave number at the peak energy $\left( {{\text{c}} = \lambda {\text{f}} = {\text{f}}/{\text{k}}} \right)$ [11]. It should be noted that there is a significant difference of the 2D FFT results between the loading frequencies of 2000 and 600 Hz (Fig. 3). Under a loading frequency 2000 Hz, the wave propagated from the driving point to the far field apparently, and the wave speed was estimated as 29.8 m/s. In contrast, under a loading frequency 600 Hz, there was no apparent wave propagation from the driving point to the far field; as a result, the wave speed could not be determined.

**Fig. 4:**
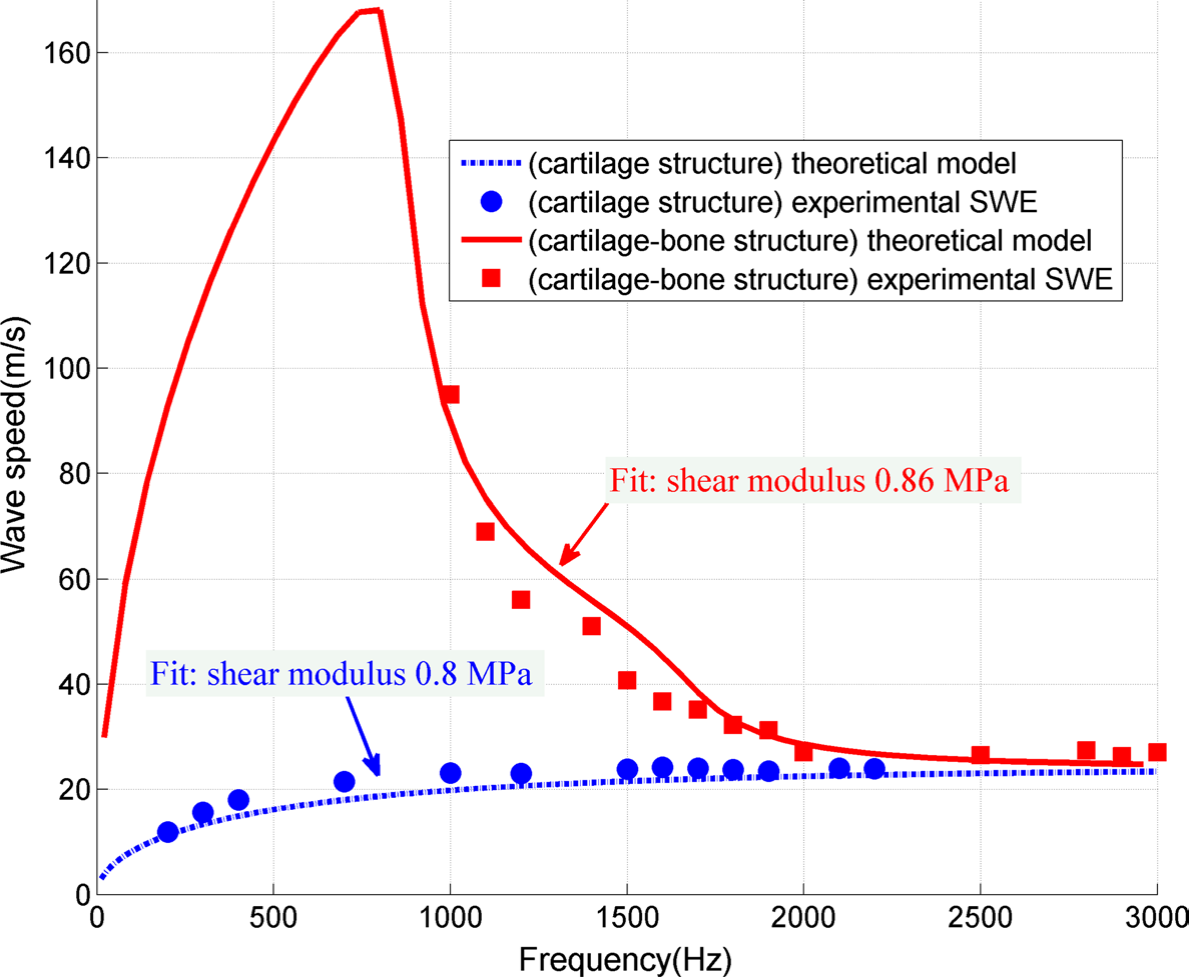
Relationship between wave speed and frequency in the pure cartilage and cartilage-bone structures. The shear modulus of silicone (simulated cartilage) was about 0.77 MPa, based on the uniaxial compression tests

The wave dispersion curve in the cartilage-bone structure (Fig. 4) was calculated by repeating the wave speed measurement process at loading frequencies ranging from 200 to 3000 Hz; the silicone elasticity (simulated cartilage) was estimated using the lamb wave model (Eq. 8). For comparison, the wave dispersion curve and elasticity measurement in the pure cartilage structure was also researched by experimental LWM and lamb wave model (Eq. 2). The silicone elasticity (simulated cartilage) is shown in the plot, the average and standard deviation is summarized in Table 1. The shear modulus was also tested by uniaxial compression tests. Good agreement is shown in Table 1 between the uniaxial compression test and LWM tests for both the pure cartilage and cartilage-bone structures.

**Table 1:** Shear modulus estimated by uniaxial compression, lamb wave method in cartilage-bone and pure cartilage structure

Method	Shear modulus (MPa)
Uniaxial compression	0.77 ± 0.18
Lamb wave method (cartilage-bone structure)	0.86 ± 0.06
Lamb wave method (pure cartilage structure)	0.80 ± 0.03

Figure 4 shows two significant difference between the pure cartilage and cartilage-bone structure. The wave dispersion curve is the first difference between the pure cartilage and cartilage-bone structure (Fig. 4). In the pure cartilage structure, the wave speed increased slowly and approached a steady value under the higher wave frequency (about 24.2 m/s). In the cartilage-bone structure, with a higher wave frequency, the wave speed increased sharply to a peak speed (about 165 m/s), then dropped to a steady value (about 25.1 m/s). Results demonstrate that the existence of bone had a significant effect on the wave speed in cartilage. Nevertheless, both the steady values in pure cartilage and cartilage-bone structure seemed to be approximately the same as the value of the estimated Rayleigh wave speed (${\text{c}} = \frac{1}{1.05}\sqrt {\mu /\rho } = 23.74 {\text{m}}/{\text{s }}$ [17]). Actually, the lamb wave speed converges to the Rayleigh wave speed as the frequency increases [18]. The frequency range of wave speed measurement is the another difference between the pure cartilage and cartilage-bone structure (Fig. 4). For the pure cartilage structure, the wave speed in the entire frequency region could be measured; however, for the cartilage-bone structure, the wave speeds could only be estimated when the frequency was higher than the critical frequency *f*_*critical*_ 1000 Hz. Two questions emerged from these observations: in cartilage-bone structure, why could the wave speed be estimated only when the frequency was higher than the critical frequency, and what may affect the critical frequency? It is crucial for the elasticity measurement in actual cartilage-bone structure.

**Fig. 5:**
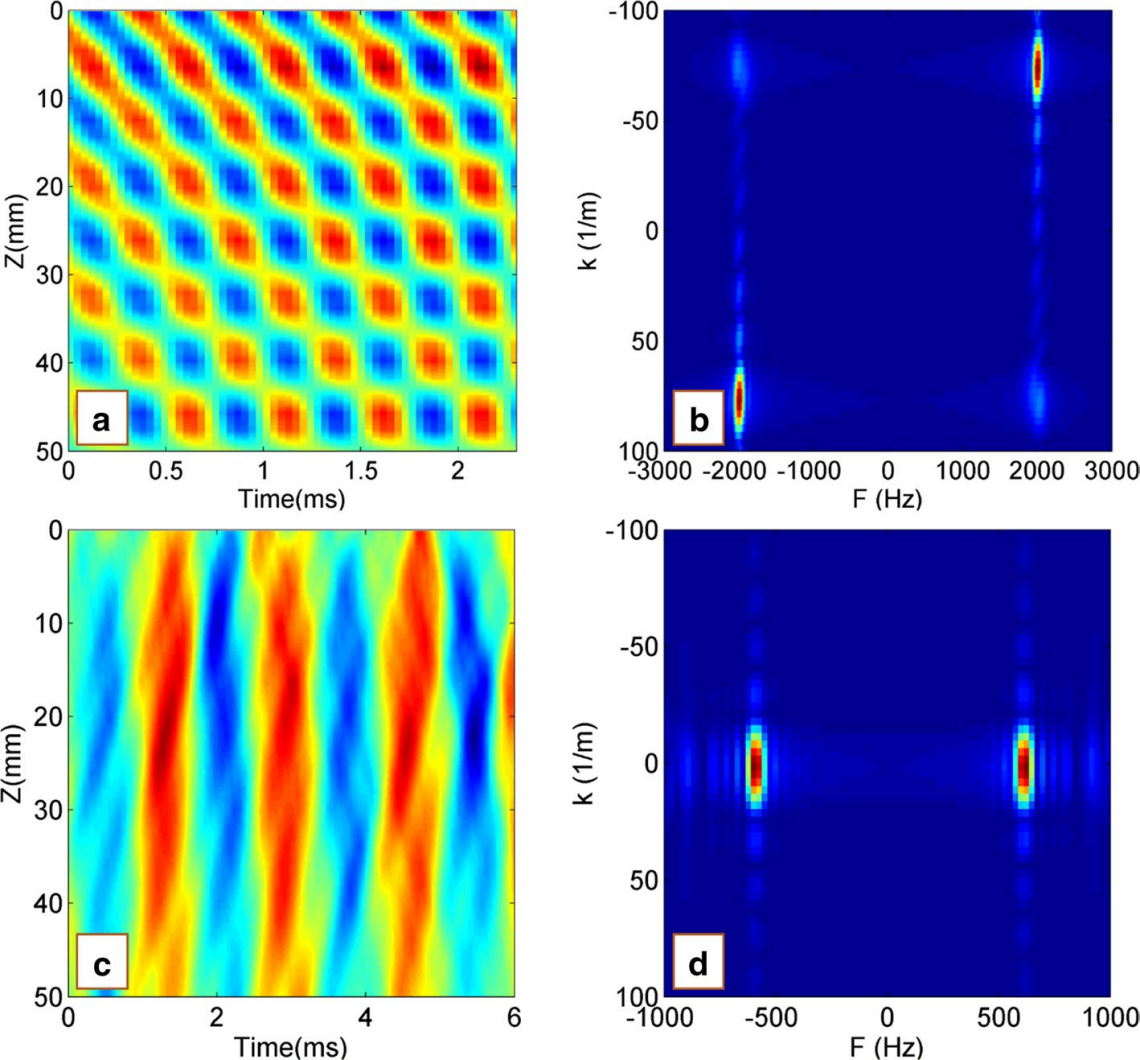
Numerical LWM results for the cartilage-bone structure: **a**, **c** velocity field of wave propagating and **b**, **d** the 2D FFT results. The wave speeds were estimated by dividing the frequency and wavenumber at the peak energy $\left( {{\text{c}} = \uplambda {\text{f}} = {\text{f}}/{\text{k}}} \right).$ **a**, **b** Loading frequency 2000 Hz, estimated wave speed 28 m/s; **c**, **d** loading frequency 600 Hz, estimated wave speed unknown

**Fig. 6:**
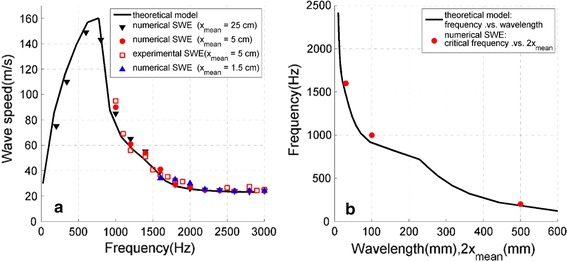
**a** Relationship between wave speed and frequency with different mean receiver distances. **b** Frequency vs. wavelength (according to the theoretical model in **a**), and critical frequency vs. double of the mean receiver distance (2x_mean_) in **a**

To further understand wave propagation in the cartilage-bone structure, numerical LWM tests were performed by finite element simulation. All the structural parameters, material parameters, and wave speed measurement methods were consistent with those of the experimental LWM. Here, the mean receiver distance was 5 cm. For comparison with the experimental results, the numerical LWM results with wave frequencies of 2000 and 600 Hz are given in Fig. 5, and the wave dispersion curve is shown in Fig. 6. The comparison indicated that the numerical LWM results were in good agreement with the experimental LWM results. The wave speed was estimated as 28 m/s with loading frequency of 2000 Hz; however, the wave speed could not be estimated with a loading frequency of 600 Hz, which was less than the critical frequency *f*_*critical*_ 1000 Hz (Fig. 5c, d). The wave speeds could only be estimated when the loading frequency was higher than the critical frequency $f_{critical}$ of 1000 Hz.

Figure 5 shows the numerical LWM results of the simulated wave propagation under a mean receiver distance 5 cm. For more in depth research, additional numerical LWM tests were performed under the following two conditions: (1) mean receiver distance *x*_*mean*_ of 25 cm; and (2) mean receiver distance *x*_*mean*_ of 1.5 cm. Figure 6a compares the relationship between the wave speed and the frequency under different mean receiver distances. It can be seen that the higher the mean receiver distance *x*_*mean*_, the lower the critical frequency. We speculate that this phenomenon may be due to the near field effect. It is well known that a near field region that is associated with the wavelength λ exists in wave propagation. When a receiver is located too close to the driving force point, wave propagation is impacted by near field effects. The task was finding a way to estimate the critical frequency theoretically. Based on the theoretical model in Fig. 6a, we estimated the frequency–wavelength curve in Fig. 6b. In addition, the numerical LWM values in Fig. 6a were also plotted in Fig. 6b as critical frequency vs. 2*x*_*mean*_. A good agreement was observed. Therefore, the receiver is located in the near field region when the wavelength $\left( {\lambda = c/f} \right)$ is larger than 2*x*_*mean*_, i.e.

10 $$f_{critical} \approx \frac{c}{{2x_{mean} }}$$

## Discussion

Elasticity measurement of simulated cartilage in cartilage-bone structure was developed using experimental LWM tests. The measured shear modulus were in good agreement with uniaxial compression tests. In addition, a near field region was found to exist in the LWM tests. When the receiver was located in the near field region, the wave speed estimated by the current method was invalid. To prevent occurrence of the near field effect and measure a valid wave speed, the wave frequency should be higher than the critical frequency. The critical frequency was about the ratio of wave speed and 2*x*_*mean*_ in current study. That is, the shorter mean receiver distance *x*_*mean*_ required a higher critical wave frequency.

The maximum Young’s modulus for the commercial SWE machine is about 0.3 MPa, and the simulated cartilage in the present study had a Young’s modulus of about 2.27 MPa. Hence, the simulated cartilage elasticity exceeded the maximum value of the commercial SWE machine, i.e., the commercial SWE machines (such as the SSI [9] or VTIQ [10]) could not be used in the present study. To study wave propagation in a cartilage-bone structure with higher elasticity, a mechanical shaker and laser Doppler detector were adopted to enhance the driving force and measurement sensitivity [19], respectively. However, this method cannot be translated to elasticity measurement of cartilage in vivo. In spite of this limitation, this method could be used to study the wave propagation model [17] and the near field effect, and could establish the theoretical foundation for the LWM in actual cartilage. Nevertheless, it remains challenging to set up the measurement machine for a cartilage-bone structure. In the future, it would be preferable to use ultrasound [7] or MRE [20] to measure wave motion. Certainly, the measurement’s accuracy and driving force should be increased [7].

The cartilage-bone structure was simulated by silicon-polypropylene plate in the present study. In terms of material properties, the actual cartilage is the porous hydrous material, and its elasticity (Young’s modulus 5.98–26.51 MPa [6]) is slightly higher than the elasticity of simulated cartilage (Young’s modulus 2.27 MPa). In terms of the structure’s shape, the actual cartilage has a curved surface and an uneven thickness. In spite some distinctions, current simulated silicon-polypropylene plate captured the main features of actual cartilage-bone structure, i.e., the actual cartilage-bone structure has a two-layered structure, and the elastic modulus of bone is much higher than cartilage. Therefore, the wave propagation model and the near field effect established in present study are reliable. The theoretical model developed in this study can also be used in LWM of actual cartilage.

Like the arterial wall and myocardium, cartilage has a “thin thickness”. Even so, there exist two important differences. First, the wave propagation models in single and two-layered structures were significant different because of the existence of bone. In a pure cartilage structure, which is similar to the arterial wall and myocardium (single-layered structure), the wave speed increased and approached the Rayleigh wave speed at a higher wave frequency [11]; in a cartilage-bone structure (two-layered structure), at a higher wave frequency, the wave speed increased sharply to a peak speed and then dropped to the Rayleigh wave speed. Secondly, the near field effect significantly affect the wave speed measurement in the cartilage-bone structure. The measured wave speed was invalid when the wave frequency was lower than the critical frequency. In contrast, this phenomenon was not observed in the pure cartilage structure (single-layered structure). The study of the critical frequency in single-layered structure is beyond the scope of this study.

The present study reveals the near field effect of LWM in a cartilage-bone structure. However, this study neglected certain factors that might influence the near field effect, and these factors should be explored in the future. First, as documented in various publications, soft tissues are inherently viscoelastic [21]. A large viscosity is linked to a large wave speed. To maintain the mean receiver distance, the critical frequency must be increased according to Eq. (10). Hence, neglecting the cartilage viscosity may underestimate the critical frequency. Second, the Poisson’s ratio could affect the longitudinal and transverse wave speed, which would change the near field region and affect the critical frequency. In future LWM studies of cartilage, the actual Poisson’s ratio of cartilage should be studied.

When the receiver was located in the near field region, a near field effect emerged in the LWM tests. It caused a deviation between the measured wave speed and the theoretical model. However, current wave inversion methods (2D FFT method) take no account of the near field effect. Hence, the wave frequency must be higher than the critical frequency to avoid the near field effect. If we have to measure the wave speed in the near field in the future, a more advanced wave inversion method that taking account of the near field effect will be required.

## Conclusion

Lamb wave method shows a potential for elasticity detection of cartilage. In this study, the elasticity measurement and wave propagation model in a cartilage-bone structure was investigated by experimental and numerical LWM tests. Results showed that the wave propagation model satisfied the lamb wave theory for a two-layered structure. Moreover, there existed the near field effect in the cartilage-bone structure. To prevent the occurrence of the near field effect and improve the validity of the wave speed measurement, the wave frequency should be higher than the critical frequency. The present study established a theoretical foundation for experimental LWM tests in cartilage, and it is the first step, to the best of the author’s knowledge, towards the ultimate goal of using LWM to diagnose osteoarthritis.
